# Single-cell atlas of urethral stricture reveals fibroblast, mast cell, and macrophage heterogeneity with candidate regulators

**DOI:** 10.3389/fimmu.2026.1932042

**Published:** 2026-10-20

**Authors:** Yue Chong, Jun Zhao, Zhenlong Wang, Delai Fu, Xiaoqiang Zhai, Linlin Zhang, Hong Wang, Mei Gao, Yu Qiao, Mingrui Li, Li Xue, Tie Chong

**Affiliations:** 1 Department of Urology, The Second Affiliated Hospital of Xi’an Jiaotong University, Xi’an, Shaanxi, China; 2 Department of Urology, The First Affiliated Hospital of Xi’an Jiaotong University, Xi’an, Shaanxi, China

**Keywords:** cell communication, cell heterogeneity, fibrosis, single-cell RNA sequencing, urethral stricture

## Abstract

**Background:**

Urethral strictures are characterized by fibrotic scar tissue formation in the urethra, leading to difficulties in urination and potential renal dysfunction. The cellular microenvironment contributes to stenosis. However, the heterogeneity of key cell types hinders our understanding of this disease.

**Methods:**

Single-cell RNA sequencing was performed on three urethral scar tissues and three normal urethral tissues. First, the main cell types and sample heterogeneity were identified. Transcription factor (TF) and enrichment analyses were used to characterize the transcriptional regulation and metabolic patterns of fibroblasts and mast cells. Finally, CellChat was used to elucidate intercellular communication networks in urethral scar tissues.

**Results:**

Of the eight cell types detected, fibroblasts, mast cells, and macrophages were major contributors to urethral scar tissue. These cells were categorized into seven (FB0–FB6), two (Mast0 and Mast1), and nine (Macro0-Macro8) subclusters, respectively, based on heterogeneity. Urethral scar tissue exhibited increased proportions of FB3 fibroblast, Mast1 mast cell, Macro1, and Macro7 Macrophages. Marker genes of these subclusters were associated with fibrosis-related biological processes, including cell adhesion and extracellular matrix organization. Additionally, screening of the dysregulation of multiple TFs and metabolic pathways revealed potential candidate TFs—ETS1 (from FB3) and SMAD3 (from Mast1)—associated with fibrosis. Furthermore, communication substantially increased between fibroblasts and other cells, and between Macro1 or Macro7 and other macrophage subclusters or fibroblasts in urethral scar tissue.

**Conclusions:**

We revealed the transcriptional characteristics of candidate cells involved in the fibrogenesis of urethral stricture, which provides insights into the pathogenesis of urethral stenosis and may facilitate the exploration of therapeutic targets.

## Introduction

Urethral stricture is a common disease of the urinary system, and its etiology includes congenital or acquired secondary factors such as iatrogenic surgery, infection, and trauma (1). It is a fibrotic luminal stenosis caused by fibrosis of the urethral mucosa and surrounding cavernous tissue, which leads to narrowing of the urethral lumen and impaired urine flow (2). The deterioration of urethral strictures can cause irreversible damage to the entire urethra. It can even induce serious complications, such as renal dysfunction, which seriously threatens the physical and mental health of patients (3). Although advances in surgical techniques have improved the success rate of urethral stricture treatment, poor long-term outcomes and high recurrence rates still pose serious challenges for clinical management (4). Unlike the clinical management of fibrosis in multiple solid organs, such as the lungs and kidneys, no drugs can effectively reverse urethral fibrosis. An emerging hypothesis suggests that postsurgical scar formation and subsequent contraction are the primary causes of urethral stricture occurrence and recurrence (5). This indicates that prevention and treatment of periurethral scarring may effectively improve the outcome of urethral stricture treatment. However, the molecular mechanisms underlying scar formation in urethral stricture remain unclear.

Multiple cell types are involved in scar formation in urethral strictures, among which fibroblasts play an important role (6). Fibroblasts are a population of cells with diverse functions, and altering the properties of fibrotic fibroblasts holds promise for overcoming the pathological progression of scars (7, 8). Therefore, exploring the heterogeneity of fibroblasts will contribute to a better understanding and treatment of fibroproliferative disorders such as urethral fibrosis. Recently, single-cell RNA sequencing (scRNA-seq) has emerged as a powerful tool for revealing the heterogeneity of fibroblasts under pathological conditions (9). It facilitates the identification of many different cell types from a sample, along with detection of complex connections and signaling mechanisms between cells (10). Two prior scRNA-seq studies have provided valuable insights into urethral stricture. Zhang et al. focused on lichen sclerosus urethral stricture and identified a unique SAA1/SAA2-expressing fibroblast subset (Fib7) and macrophage M1/M2-like imbalance using 16, 937 cells from three LS USD and three non-LS USD samples (11). Li et al. analyzed post-traumatic urethral stricture (PTUS) and described fibroblast lineage-specific regulatory changes and integrin-mediated fibroblast–endothelial communication using 13, 411 cells (12). These studies established the importance of fibroblast heterogeneity and immune–stromal crosstalk in specific urethral stricture subtypes. However, both were based on relatively small cell numbers and focused on a single etiology; the mast cell compartment, metabolic reprogramming, and integrated transcription factor–metabolism–communication networks remain largely unexplored. In addition, the two studies primarily emphasized fibroblasts, endothelial cells, and macrophages, whereas a systematic comparison of fibroblast, mast cell, and macrophage heterogeneity within the same urethral stricture dataset has not been reported.

In this study, we employed scRNA-seq analysis of urethral scar and normal tissues to identify major cell populations. In particular, we describe the transcriptional features and regulatory signals of fibroblasts. Mast cells, as inflammatory cells, can stimulate fibroblasts to release mediators of scar formation via paracrine mechanisms (13). Macrophages are key components of the innate immune system and are involved in antigen presentation, inflammatory response, and tissue repair during healing (14). Thus, we also performed transcriptional characterization of mast cell and macrophage subtypes. This study provides insights into the pathological mechanisms of urethral stricture and provides a useful reference for developing new therapeutic strategies.

## Materials and methods

### Collection of clinical specimens

Urethral scar tissues (BH group) and paired normal urethral tissues (ZC group) were collected from three patients with urethral stricture who were admitted to our hospital. The clinical characteristics of the patients are summarized in **Table 1**. The study was approved by the Clinical Ethics Committee of the Second Affiliated Hospital of Xi’an Jiaotong University, and written informed consent was obtained from all patients. All procedures were conducted in accordance with the guidelines of the Declaration of Helsinki.

**Table 1:** Clinical data of single-cell sequencing samples of three patients with urethral stricture.

Donor	Gender	Age (years)	History	Stricture location	Pathological results	Preoperative time	Surgical treatment
Patient 1	Male	53	Trauma	Penile urethra	Pathological diagnosis	5 months	Urethroplasty
Patient 2	Male	46	Trauma	Bulbar urethra	Pathological diagnosis	5 months	Urethroplasty
Patient 3	Male	19	Trauma	Bulbar urethra	Pathological diagnosis	3 months	Urethroplasty

### Sample preparation for scRNA-seq

Tissue specimens were washed with ice-cold RPMI1640 and digested with various digestive enzymes (DNase I [Absin, abs47048001], collagenase II [Absin, abs47048001], and collagenase IV [Absin, abs47047435]) for 30 min at 37 °C. Erythrocytes were then removed with erythrocyte lysate (Biosharp, BL503A). Next, the AO/PI reagent (Miltenyi, 130-090-101) cell suspensions were analyzed using a fluorescent cell analyzer to confirm the complete removal of dead cells. Finally, the cell samples were resuspended in PBS (containing 0.04% BSA solution) for subsequent scRNA-seq analysis.

### scRNA-seq library preparation and sequencing

Single-cell suspensions from each sample were processed for scRNA-seq using the Single Cell 3′ whole-transcriptome amplification protocol on the BD Rhapsody platform. Single-cell cDNA libraries were generated as previously described (15). Quantitative analysis of the PCR products was then conducted using the Qubit dsDNA HS Assay (Thermo Fisher Scientific, Q32854) according to the manufacturer’s guidelines. Finally, the resultant libraries were sequenced on an Illumina Novaseq 6000 system, with a 150 nt paired end.

### Raw sequencing data preprocessing and analysis

Raw scRNA-seq data were analyzed using R software (version 4.3.0). We first performed quality control using the Seurat R package (16). Briefly, cells with unique molecular identifier (UMI) counts < 1000, genes < 500, and mitochondrial gene fractions > 25% were discarded. Genes expressed in <5 cells were also excluded from the analysis. Next, the UMI count matrix was log-normalized using the Seurat package. For each sample, the top 2000 highly variable feature genes were selected using the findinintegrationanchors function in Seurat, followed by principal component analysis to reduce data dimensionality. The top 50 principal components were selected for downstream analysis. At a resolution of 0.6, the FindClusters function in Seurat was applied to identify cell clusters, which were then visualized using UMAP plots. Furthermore, we employed the FindAllMarkers function in Seurat to explore the genetic markers of each cell cluster. Marker genes for urethral fibrosis were also searched using ScType tools (17) and previously published articles (18–20).

### Differential expression and enrichment analyses

Differential expression of cell populations in the BH and ZC groups was analyzed using the Seurat package FindAllMarkers or FindMarkers functions. Statistical significance was determined using the Wilcoxon rank-sum test with Bonferroni correction. To further evaluate the biological functions of genes, GO and KEGG pathway analyses were performed using the KOBAS software (version 2.0) (21).

### Transcription factor regulatory network establishment

The TF-target gene network was constructed using the SCENIC Python workflow (version 0.11.2) with default parameters (22). Human TF data were extracted from the pySCENIC website (https://github.com/aertslab/pySCENIC/tree/master/resources). Activated TFs were screened using the area under curve (AUC) matrix, followed by selection of differentially expressed TFs within the BH and ZC groups using the R package limma (23). Moreover, a regulatory specificity score was assigned to the discovered regulons of each cell to identify cluster-specific regulation (24). Finally, the transcriptional regulatory network of key TFs was visualized using Cytoscape (version 3.9.1) (25).

### Metabolic pathway analysis

To reveal the complex metabolic landscape within the urethral scar, scMetabolism was utilized to quantify and visualize the metabolic diversity of single cells (26). Briefly, the activity of each cell was scored using the VISION algorithm for metabolism. Next, the activity scores of metabolic pathways in each cell cluster were comprehensively analyzed.

### Cellular communication analysis

To elucidate the potential connections between different cell types, we employed CellChat (Version 2.1.1) for cell-cell interaction analysis (20). First, the major cell types and gene expression patterns of the ZC and BH groups were entered into CellChat. Secreted signaling pathways were subsequently set up as a reference database for ligand-receptor interactions. Multiple functions, including computeCommunProbPathway and aggregateNet, were applied to calculate communication probabilities of ligand/receptor and cell/cell communication, and then infer biologically meaningful cell-cell communication networks mediated by ligand-receptor pairs.

### Immunofluorescence staining assay

Urethral scar tissues (BH group) and paired normal urethral tissues (ZC group) from another five patients were collected for immunofluorescence staining. Tissue sections were deparaffinized and rehydrated by immersion in xylene and ethanol, followed by antigen retrieval. Serum blocking was performed using 10% donkey serum for goat-derived primary antibodies or 3% BSA for other sources, followed by incubation for 30 min. The sections were then incubated with a mixture of primary antibodies (anti-a-SMA, ServiceBio, GB12045, 1:5000; anti-L1CAM, ServiceBio, GB112046, 1:800; anti-Traptase, ServiceBio, GB15104, 1:500; anti-TNFRSF9, ABMART, PK52743S, 1:100; anti-CD68, ServiceBio, GB113150, 1:5000; anti-IL-1β, ServiceBio, GB11113, 1:400; anti-CD163, ServiceBio, GB113152, 1:100; anti-TAGLN, ServiceBio, GB11366, 1:1500) overnight at 4 °C. After washing with PBS, the corresponding fluorescently labeled secondary antibodies were added and the cells were incubated at room temperature for 50 min. The nuclei were counterstained with DAPI for 10 min in the dark. Following autofluorescence quenching, sections were mounted with an anti-fade medium. Finally, images were captured using a NIKON ECLIPSE C1 fluorescence microscope (Nikon, Japan) at specific wavelengths: DAPI for blue, 488 for green, CY3 for red, and CY5 for yellow-positive signals. Images were processed and quantified using ImageJ software (NIH, USA). The fraction of L1CAM-positive area (%) was calculated as the ratio of the L1CAM-positive area to the total tissue area. The mean fluorescence intensity (A.U.) of Tryptase, TNFRSF9, IL-1β, CD68, CD163, and TAGLN were measured in five randomly selected fields per section.

### Toluidine blue staining

Toluidine blue staining, a metachromatic staining method in which mast cells appear red or purple (metachromatic) and the background stains blue (orthochromatic), was employed for mast cell identification. Briefly, 5-µm thick tissue sections mounted on glass slides were rehydrated. These were then stained with 0.1% toluidine blue for 3 min. Next, the sections were rinsed with distilled water, dehydrated in ethanol, cleared in xylene, and mounted using the Krystalon reagent (EMD Millipore, MA, USA). For quantitative analysis, mast cells stained purple-red were counted in five randomly selected fields (40×) per section.

### Statistical analysis

Statistical significance was determined using the Wilcoxon rank-sum test with the Bonferroni correction for differential expression analysis. The thresholds for differentially expressed genes (DEGs) were as follows: difference in percentage of detected cells ≥ 0.15, natural log scale ≥ 0.5, adjusted p < 0.05. For functional enrichment analysis, Benjamini–Hochberg-corrected p < 0.05 was adopted as the result of significant enrichment. For patient-level pseudobulk analysis, cells were aggregated per sample and cell type, and differential expression was analyzed using DESeq2 with a paired design (~ patient + group). For histological and immunofluorescence quantification, data are presented as the mean ± SD. Differences between the two groups were analyzed using a paired Student’s t-test. The histological and immunofluorescence quantifications were performed by an observer blinded to group assignment. A *p*-value < 0.05 was considered statistically significant. All statistical analyses were performed using GraphPad Prism (version 10.0).

## Results

### Single-cell landscape of BH and ZC groups

To comprehensively characterize the specific gene expression profiles of each cell type in urethral fibrosis, we collected three urethral scar tissue samples (BH group) and three paired normal urethral tissue samples (ZC group) from patients with urethral strictures for scRNA-seq analysis (**Figure 1A**). After the initial quality control, 65, 054 single cells were obtained for subsequent analysis. The cells were clustered using the UMAP algorithm, resulting in 27 clusters. The top three specifically expressed marker genes (top three) for these clusters are shown in **Figure S1A**. Eight cell categories were successfully annotated based on the unique marker gene expression. These included B cells (with the marker genes JCHAIN, CD79A, and MS4A1), endothelial cells (ACKR1, HMOX1, and CLDN5), epithelia (KRT5, KRT13, and KRT15), fibroblasts (DCN, APOD, FBLN1, and COL3A1), macrophages (LYZ, TYROBP, CD163, and S100A4), mast cells (TPSB2, CPA3, MS4A2, and KIT), SMC (ACTA2, RGS5, and MYH11), and T cells (PTPRC, TRAC, and CD3E) (**Figure 1C**). All cell types were detected in the ZC and BH groups but exhibited different distribution patterns. The populations of endothelial cells, fibroblasts, mast cells, and SMCs in the BH group increased compared with those in the ZC group, whereas those of epithelia, macrophages, B cells, and T cells decreased (**Figure 1D**). We also observed differences in the relative number of cells between the two groups; the proportion of mast and epithelial cells showed the greatest changes compared to that in the ZC group (**Figure 1E**). Furthermore, GO terms were enriched in the top 50 marker genes in each cell type (**Figure 1F**). For instance, fibroblast marker genes were enriched in extracellular matrix (ECM) and collagen fibril organization pathways, which play essential roles in the progression of urothelial fibrosis (27). Marker genes of mast cells were enriched in immune and inflammatory responses. The marker genes of macrophages were involved in immunoglobulin production, which is involved in the immunoglobulin-mediated immune response and peptide antigen assembly with the MHC class II protein complex. Based on the eight cell types, 822 DEGs (574 upregulated and 248 downregulated) were screened between the BH and ZC groups. Most of these were distributed in endothelial cells, mast cells, epithelia, and SMC (**Figure S1B**). Further GO enrichment analysis revealed that the DEGs whose expression was upregulated in most cell types, such as fibroblasts, endothelial cells, SMC, and mast cells, were mainly related to the negative regulation of cell population proliferation, cellular response to heat, and response to unfolded proteins (**Figure S1C**). DEGs with downregulated expression in cells such as epithelia were mainly enriched in intermediate filament organization (**Figure S1D**). To account for potential pseudo-replication, we performed patient-level pseudobulk analysis, which confirmed the concordance of the main single-cell findings (**Supplementary Figure S7**). Taken together, these results suggest that the BH and ZC groups exhibit distinct single-cell transcriptome patterns. Changes in the number of fibroblasts, mast cells, and macrophages were observed in the BH group, suggesting that these cells promote fibrosis in urethral scar tissue.

**Figure 1:**
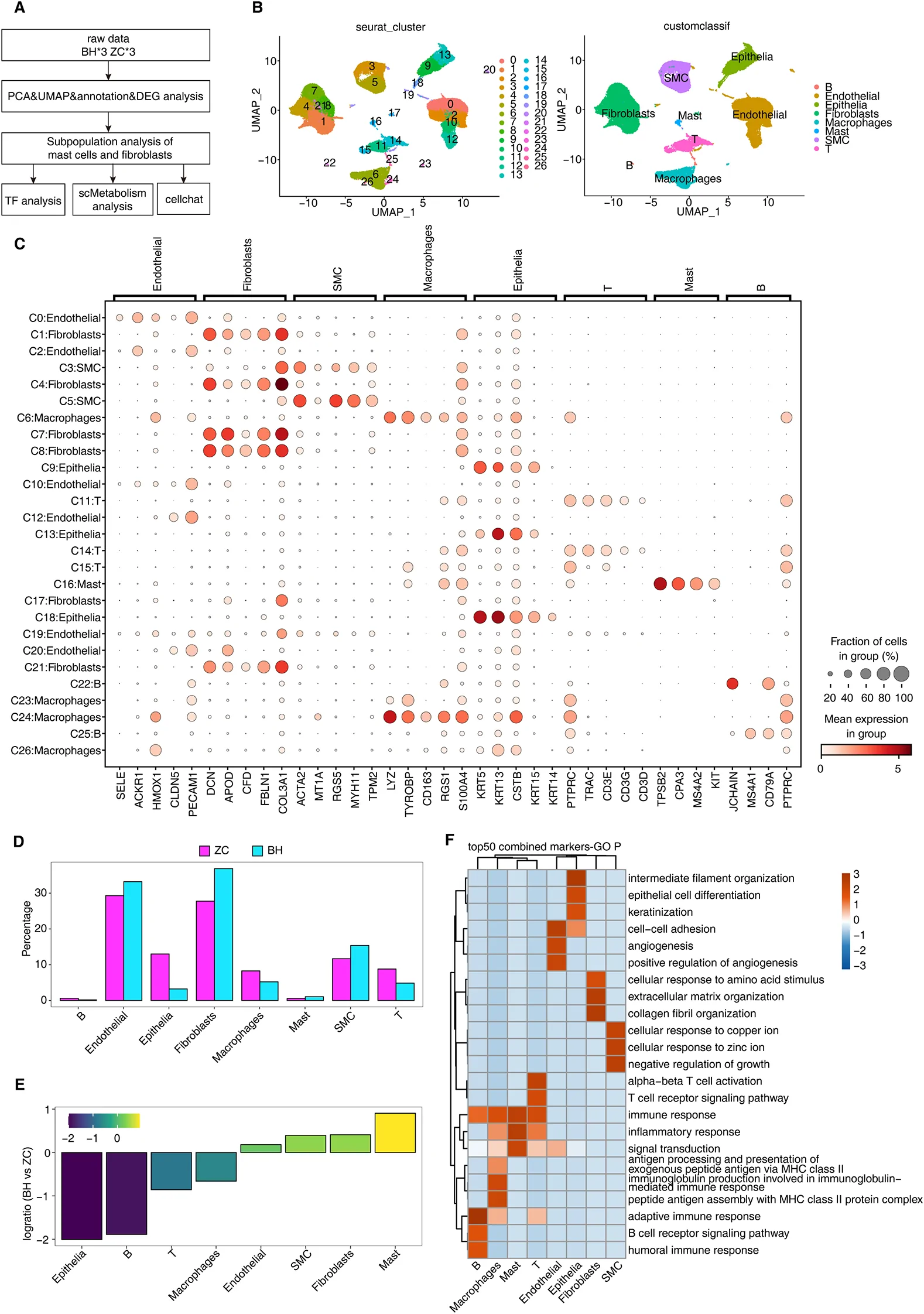
Single-cell atlas of urethral scar (BH) and normal urethral (ZC) tissue samples. **(A)** Schematic of the workflow. **(B)** UMAP plot of 27 clusters (left) assigned to eight known cell categories (right) based on the marker gene expression. **(C)** A dot plot of representative marker genes for each cell population. **(D)** Proportions of each cell type in the BH and ZC groups. **(E)** Relative change values for each cell type in the BH versus ZC groups. **(F)** A heatmap showing the GO biological processes enriched for the top 50 marker genes in each cell cluster. Three patients with paired scar (BH) and normal (ZC) tissues were analyzed.

### Characterization of fibroblast heterogeneity

As fibroblasts are the most abundant cell type in scar tissue, we further explored the role of their heterogeneity in urethral fibrosis. We extracted all fibroblasts and performed unsupervised cluster analysis. Finally, seven fibroblast subclusters were identified, ranging from FB0 to FB6 (**Figure 2A**). **Figure 2B** shows the proportions of fibroblast subclusters in the BH and ZC groups. The proportions of FB1, FB2, and FB3 increased in the BH group compared to that in the ZC group, whereas the proportion of the remaining subtypes decreased. **Figure 2C** shows the top marker genes for each fibroblast subcluster, together with the canonical fibroblast markers (COL1A1, COL1A2, and COL3A1). These canonical markers were expressed in the fibroblast subclusters, supporting their fibroblast identity. FB1 highly expressed GSN and NTRK2; FB2 highly expressed TIMP1 and APOD; and FB3 highly expressed PLP1, CDH19, and L1CAM. The immunofluorescence results showed that L1CAM exhibited a prominent red fluorescence signal in fibrotic tissues, which was almost absent in normal tissues (7.21 ± 2.92% vs. 2.15 ± 0.95%, p < 0.01, **Figure 2D**). L1CAM was co-localized with α-SMA, suggesting a potential association with fibrosis. Further, GO analysis indicated that the top 100 marker genes of the FB3 subcluster were closely associated with key fibrosis-related biological processes, including ECM organization and cell adhesion (**Figure 2E**).

**Figure 2:**
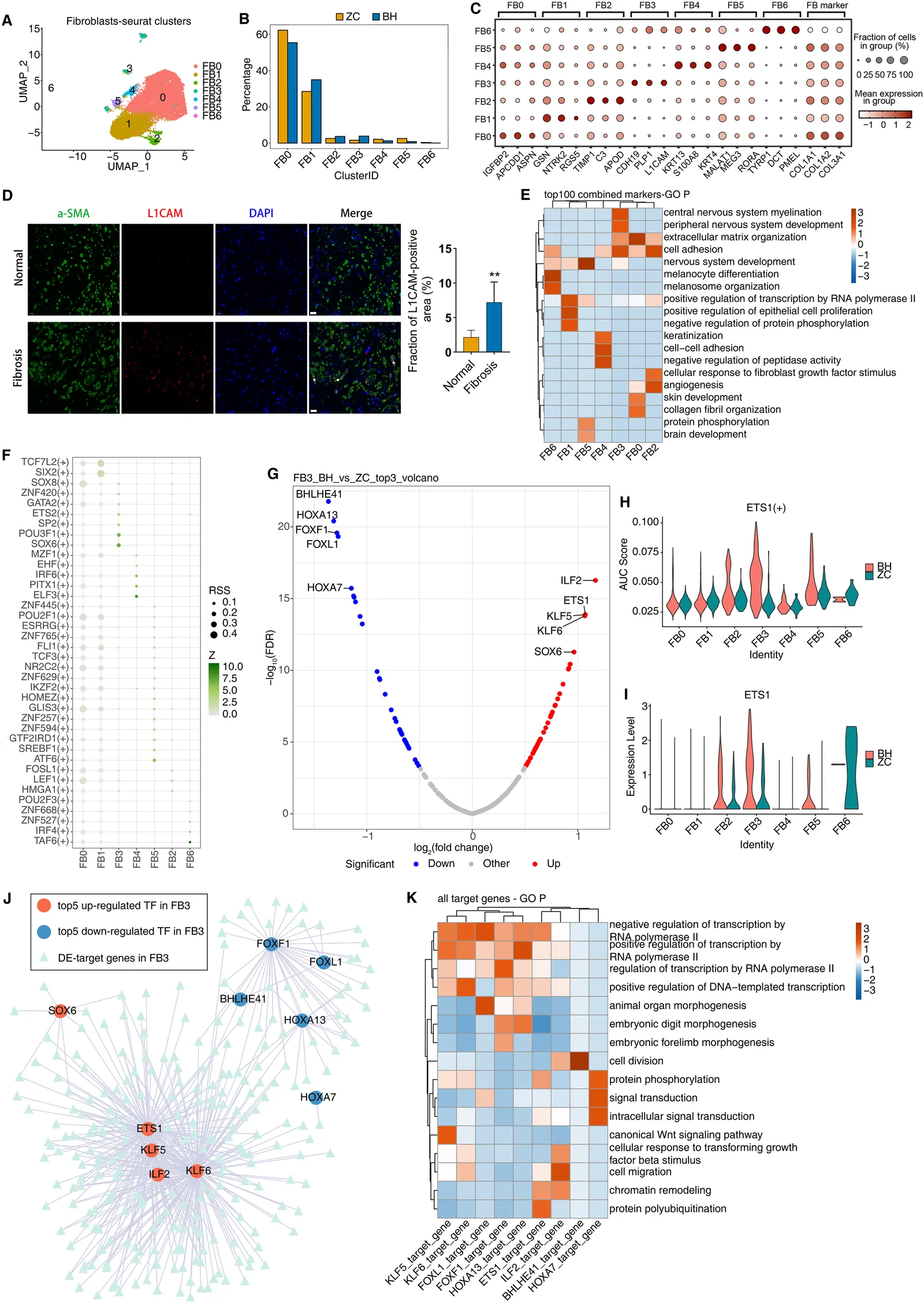
Transcriptomic heterogeneity of fibroblasts. **(A)** UMAP of the seven fibroblast subclusters [FB0–FB6]. **(B)** Proportions of FB0–FB6 cells in the BH and ZC groups. **(C)** A dot plot showing the top marker genes for each fibroblast subcluster, together with canonical fibroblast markers (COL1A1, COL1A2, and COL3A1). **(D)** Immunofluorescence staining showing the localization and expression of α-SMA and L1CAM in normal and fibrosis tissues. The fraction of L1CAM-positive area (%) was quantified. **(E)** A heatmap showing GO enrichment terms of the top 100 marker genes in each fibroblast subcluster. **(F)** A dot plot of specific TF regulons in different fibroblast subclusters. **(G)** A volcano plot displaying differentially expressed TFs within the FB3 subcluster from the BH and ZC groups. Red and blue dots represent TFs with upregulated and downregulated expression, respectively. **(H, I)** A violin plot showing the AUC score and expression level of ETS1 in each fibroblast subcluster of different samples. **(J)** The TF-target gene regulatory network in the FB3 subcluster. The top five TFs with upregulated and downregulated expression are selected. A square represents TF, and a triangle represents target gene. **(K)** A heatmap illustrating the GO enrichment results for target genes of TFs in the regulatory network. For scRNA-seq, three patients with paired tissues were analyzed. For immunofluorescence, five patients with paired tissues were analyzed, and five randomly selected fields per section were quantified. Data are presented as mean ± SD. Statistical significance was determined using a paired Student’s t-test. ** *p* < 0.01.

To understand the transcriptional state of the cells, we employed SCENIC to identify the TFs involved in the regulation of fibroblast subclusters. TCF7L2 and SIX2 were enriched in FB0; FOSL1, LEF1, and HMGA1 expression was upregulated in FB2; and ETS2, POU3F1, and SOX6 were markedly elevated in FB3 (**Figure 2F**). TFs such as HMGA1, FOSL1, ETS2 and SOX6, are involved in fibrosis regulation, suggesting that these specific TFs may facilitate fibrosis (28–31). Given that the proportion of the FB3 subcluster showed the strongest tendency to increase in the BH group, we identified TFs that were differentially expressed between the BH and ZC groups. We observed clear changes in the expression of multiple TFs. The volcano plot illustrates the top 50 differential TFs; the expression of ETS1, KLF5, KLF6, and SOX6 was significantly upregulated in the BH group, whereas that of HOXA13 and FOXF1 was downregulated (**Figure 2G**). The AUC score and expression levels further supported the aberrant changes in these TFs in the disease (**Figures 2H, I**; **Supplementary Figures S2A–J**). A regulatory network based on differential TFs (top five upregulated and downregulated) and DEGs in the FB3 subcluster (**Figure 2J**) was established. GO enrichment analysis indicated that the target genes of several factors (e.g., KLF5, KLF6, FOXL1, FOXF1, and ETS1) were principally involved in the regulation of transcription by RNA polymerase II-related terms and protein polyubiquitination; targets of HOXA7 were mainly enriched in signal transduction-related terms, and targets of BHLHE41 were closely associated with cell division (**Figure 2K**).

Because the number of FB0 fibroblasts was reduced in the BH group, we also explored the transcriptional regulatory mechanisms. First, we analyzed the differentially expressed TFs in the FB0 subcluster. Key TFs that were aberrantly expressed in FB3 cells, such as KLF5, KLF6, ILF2, and BHLHE41, were similarly altered in FB0 cells (**Supplementary Figures S2K–O**). The TF-target gene network showed that these TFs regulated the expression of multiple target genes, especially KLF6 (**Supplementary Figure S2P**). Moreover, GO enrichment analysis indicated that most target genes were primarily enriched in the regulation of transcription by RNA polymerase II-related categories, which was consistent with the results for FB3 (**Supplementary Figure S2Q**). These findings further suggest that these TFs may play potential regulatory roles in the progression of fibrosis.

### Analysis of metabolic pathways in different fibroblast subtypes

Abnormal metabolic patterns may be important factors in the development of urethral fibrosis (32). Therefore, we further explored the specific metabolic pathways of each fibroblast subcluster. As shown in **Figure 3A**, cysteine and methionine metabolism, oxidative phosphorylation, and tyrosine metabolism were significantly enriched in the FB0 subcluster. Glycine, serine, and threonine metabolism; fatty acid elongation; and lysine degradation were mainly activated in the FB3 subcluster. We focused on the differential metabolic pathways in BH and ZC in the FB3 subcluster. Porphyrin and chlorophyll metabolism was significantly upregulated in the BH group, whereas arachidonic acid metabolism, metabolism of xenobiotics by cytochrome P450, and drug metabolism-cytochrome P450 were downregulated (**Figure 3B**). Changes in the activity of these metabolic pathways were also observed in other fibroblast subclusters (**Figures 3C–F**).

**Figure 3:**
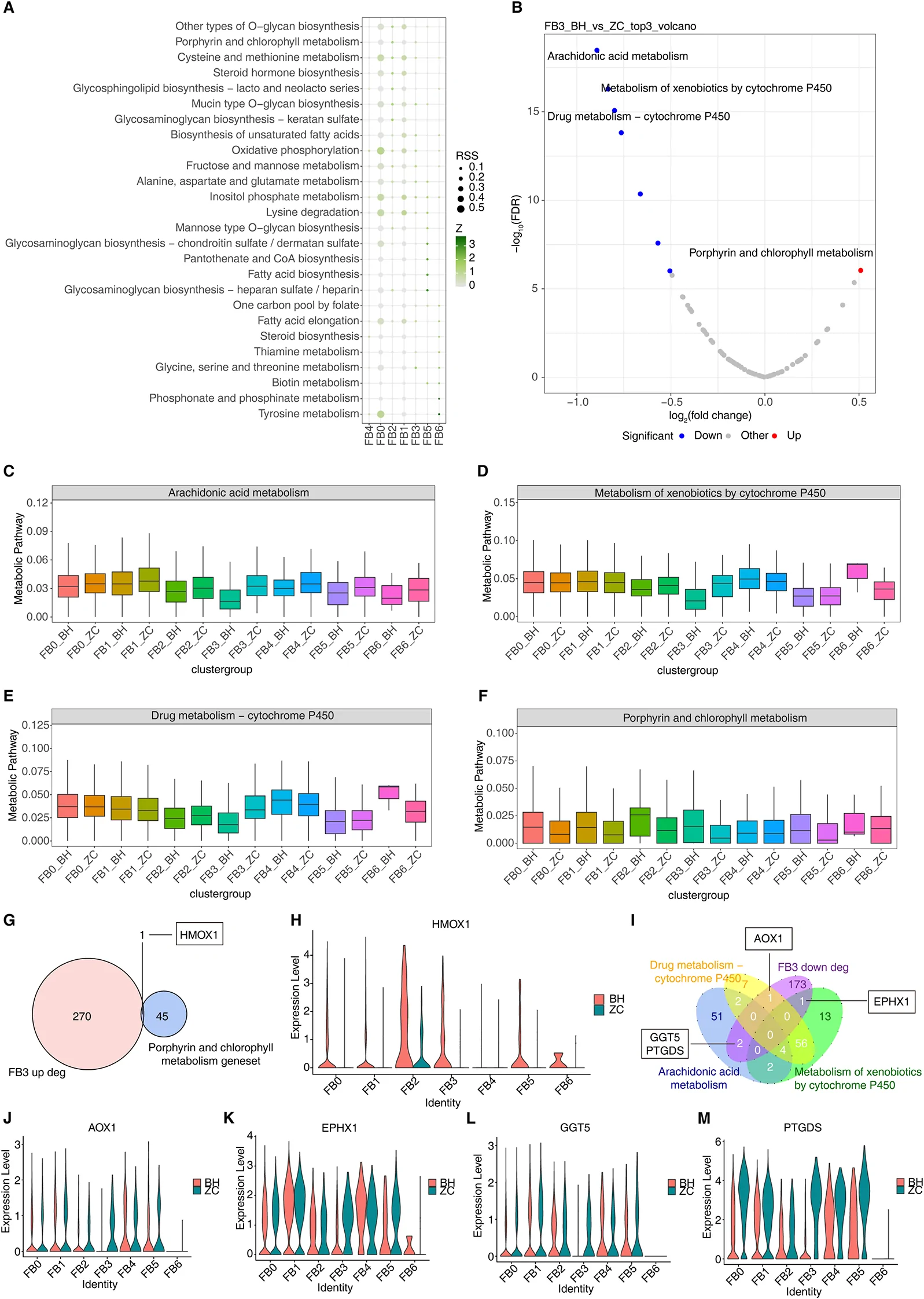
Comparison of metabolic pathways of the FB3 subcluster between the BH and ZC groups. **(A)** A dot plot showing specific metabolic pathway scores in different subclusters of fibroblasts. **(B)** A volcano plot of differential metabolic pathways in the FB3 subcluster from the BH and ZC groups. **(C–F)** Box plots showing the scores of arachidonic acid metabolism, metabolism of xenobiotics by cytochrome P450, drug metabolism-cytochrome P450, as well as porphyrin and chlorophyll metabolism in fibroblast subclusters. **(G)** A Venn diagram of DEGs with upregulated expression in the FB3 subcluster and gene set in porphyrin and chlorophyll metabolism. **(H)** Expression levels of HMOX1 in seven fibroblast subclusters. **(I)** A Venn diagram of DEGs with downregulated expression in the FB3 subcluster and gene sets in three downregulated metabolic pathways. **(J–M)** Expression levels of AOX1, EPHX1, GGT5, and PTGDS in seven fibroblast subclusters. Three patients with paired scar (BH) and normal (ZC) tissues were analyzed.

To explore the genes involved in the regulation of these metabolic pathways, the gene sets of metabolic pathways were integrated with DEGs in the FB3 subcluster. HMOX1 expression during porphyrin and chlorophyll metabolism was upregulated in FB3 cells (**Figures 3G, H**). However, the expression of GGT5 and PTGDS in arachidonic acid metabolism, EPHX1 expression in the metabolism of xenobiotics by cytochrome P450, as well as AOX1 expression in drug metabolism-cytochrome P450, were downregulated in FB3 cells (**Figures 3I–M**). In addition, HMOX1 expression in porphyrin and chlorophyll metabolism was upregulated in the FB2 subcluster of the BH group (**Supplementary Figures S3A, B**).

### Characterization of mast cell heterogeneity

Considering the increased mast cell count in the BH group and its contribution to fibrosis, we performed a heterogeneity analysis of these cells. Two distinct cell subclusters—Mast0 and Mast1—were annotated based on the expression of cell markers (**Figure 4A**). The number of Mast1 subclusters decreased in the BH group compared to that in the ZC group, whereas that of the Mast1 subcluster increased (**Figure 4B**). The Mast0 subcluster highly expressed the marker genes HSPA1B, FOS, and JUN, which may reflect a stress-responsive phenotype, while the Mast1 subcluster highly expressed MT1G, TNFRSF9, and MT1X, potentially indicating a metal-ion-responsive phenotype (**Figure 4C**). Toluidine blue staining showed that the number of mast cells in fibrotic tissues was higher than that in normal tissues (*p* < 0.01, **Figures 4D, E**). Furthermore, immunofluorescence staining showed that the expression of Tryptase (*p* < 0.05) and TNFRSF9 (*p* < 0.01) in the Mast1 subcluster of fibrotic tissues was increased compared to that in normal tissues (**Figures 4F, G**). Next, we performed GO enrichment analysis of the top 100 marker genes in the two mast cell subclusters. As shown in **Figure 4H**, Mast0-derived genes were mainly enriched in the regulation of transcription by RNA polymerase II-related terms, whereas Mast1-derived genes were closely related to the cellular response to copper/zinc ions and negative regulation of growth.

**Figure 4:**
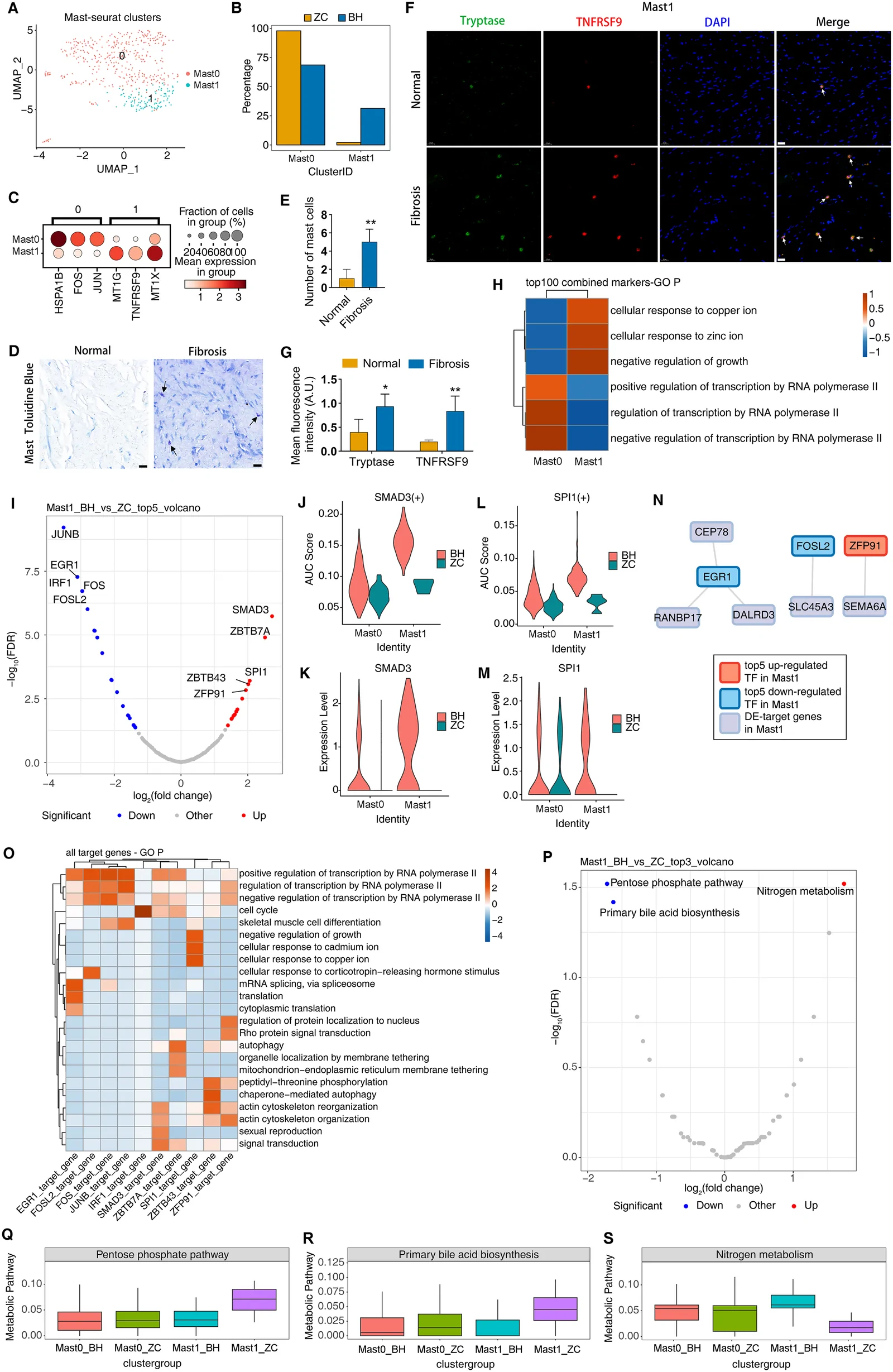
Transcriptomic heterogeneity of mast cells. **(A)** UMAP of the two mast cell subclusters [Mast0 and Mast1]. **(B)** A bar plot displaying the proportions of two mast cell subclusters in the BH and ZC groups. **(C)** A dot plot of the expression level of the top three marker genes for the Mast0 and Mast1 subclusters. **(D, E)** Toluidine Blue staining showing the distribution of mast cells in normal and fibrotic tissues, with the number of mast cells quantified. **(F, G)** Immunofluorescence staining showing the localization and expression of Tryptase and TNFRSF9 in the Mast1 subcluster from normal and fibrotic tissues, with their mean fluorescence intensity quantified. **(H)** A heatmap showing differences in GO biological processes between two mast cell subclusters. **(I)** A volcano plot of differentially expressed TFs of the Mast1 subcluster from the BH and ZC groups. **(J–M)** Violin plots showing the AUC score and expression level of SMAD3 or SPI1 in different samples from each mast cell subcluster. **(N)** The TF-target gene regulatory network in the Mast1 subcluster. The top five TFs with upregulated and downregulated expression are selected. The square represents TF and the triangle represents target gene. **(O)** GO analysis for biological processes enriched in TF-regulated target genes. **(P)** A volcano plot exhibiting the differential metabolic pathway of the Mast1 subcluster between the BH and ZC samples. **(Q–S)** Box plots displaying the activity score of three metabolic pathways (pentose phosphate pathway, primary bile acid biosynthesis, and nitrogen metabolism) of all mast cell subclusters for the BH and ZC groups. For scRNA-seq, three patients with paired tissues were analyzed. For immunofluorescence and Toluidine blue staining, five patients with paired tissues were analyzed, and five randomly selected fields per section were quantified. Data are presented as mean ± SD. Statistical significance was determined using a paired Student’s t-test. *p < 0.05, **p < 0.01.

We analyzed specific TFs in the Mast0 and Mast1 subclusters to investigate the role of TFs in mast cells in the pathogenesis of urothelial fibrosis. The TFs in these subclusters showed different expression patterns (**Supplementary Figure S4A**). TFs, such as FOS, JUNB, ATF3, JUN, and KLF2, were specifically expressed in Mast0, whereas ELF4, RFX2, ZNF777, SMAD3, and SPI1 were uniquely expressed in Mast1. In addition, we detected differentially expressed TFs in the Mast1 subcluster because of its increased proportion in the BH group. As expected, we identified several key TFs that were aberrantly expressed in the BH group. For example, the expression of SMAD3 and SPI1 was upregulated in the BH group, whereas that of JUNB and FOS was downregulated (**Figure 4I**). AUC and expression analyses further confirmed the increased expression of SMAD3 and SPI1 in the BH group (**Figures 4J-M**). We also established a TF-target gene network based on DEGs in the Mast1 subcluster; the TF EGR1 regulated the expression of multiple target genes, including CEP78, RANBP17, and DALRD3 (**Figure 4N**). Moreover, several target genes of TFs (e.g., SPI1, SMAD3) were involved in autophagy, signal transduction, the cell cycle, and the regulation of transcription by RNA polymerase II (**Figure 4O**). We further assessed the metabolic differences between BH and ZC in the Mast1 subcluster. Compared with the ZC group, nitrogen metabolism was activated in the BH group, whereas the pentose phosphate pathway and primary bile acid biosynthesis were suppressed (**Figure 4P**). These findings were supported by the activity scores of the metabolic pathways (**Figures 4Q–S**).

### Characterization of macrophage heterogeneity

Considering the decreased number of macrophages in the BH group and their contribution to fibrosis, we analyzed the heterogeneity of these cells. Nine distinct macrophage subclusters were identified based on the expression of cell markers, ranging from Macro0 to Macro8 (**Figure 5A**). The percentages of Macro1 and Macro7 were remarkably increased in the BH group compared to those in the ZC group (**Figure 5B**). The expression of the top marker genes for each macrophage subcluster and canonical macrophage markers (CD86, MRC1, and CD163) is shown in **Figure 5C**. These canonical markers were expressed across macrophage subclusters, confirming their macrophage identity. Macro1 highly expressed CXCL3, IL1B, HLA-DRB6, CXCL2, and NLRP3, which may indicate a pro-inflammatory monocyte-like phenotype; Macro7 highly expressed ACTA2, RGS5, TAGLN, COL1A1, and MYL9, suggesting an SMC-like/myofibroblast-like identity. The remaining macrophage subclusters (Macro0, Macro2–Macro6, and Macro8) also expressed distinct marker gene sets, suggesting additional functional heterogeneity within the macrophage compartment (**Figure 5C**). The top50 marker genes in Macro7 are listed in **Figure 5D**. Immunofluorescence staining showed that the expression of CD68 (*p* < 0.01) and IL-1β (*p* < 0.05) in Macro1 from fibrotic tissues was remarkably increased compared to that in normal tissues (**Figure 5E**); the expression of CD163 (*p* < 0.01) and TAGLN (*p* < 0.01) was higher in Macro7 from fibrosis tissues (**Figure 5F**). These results suggest that Macro1 and Macro7 levels were increased in scar tissue and may be associated with fibrosis. Further, GO enrichment analysis revealed that the top 50 marker genes in the Macro7 subcluster were mainly enriched in ECM organization and angiogenesis (**Figure 6A**). Additionally, the expression levels of COL1A1, COL1A2, COL3A1, THBS1, ANXA2, and S100A4, in the Macro1 and Macro7 subclusters significantly increased in the BH group compared to those in the ZC group, whereas the expression of NR4A2 and IGF1 in the Macro1 and Macro7 subclusters significantly decreased in the BH group (*p* < 0.05). CXCL8 expression in Macro1 cells was markedly upregulated in the BH group, whereas KLF2 expression in Macro1 cells was significantly decreased (*p* < 0.05). However, no significant differences in CXCL8 and KLF2 expression in the Macro7 subcluster were observed between the two groups (**Figures 6B–K**).

**Figure 5:**
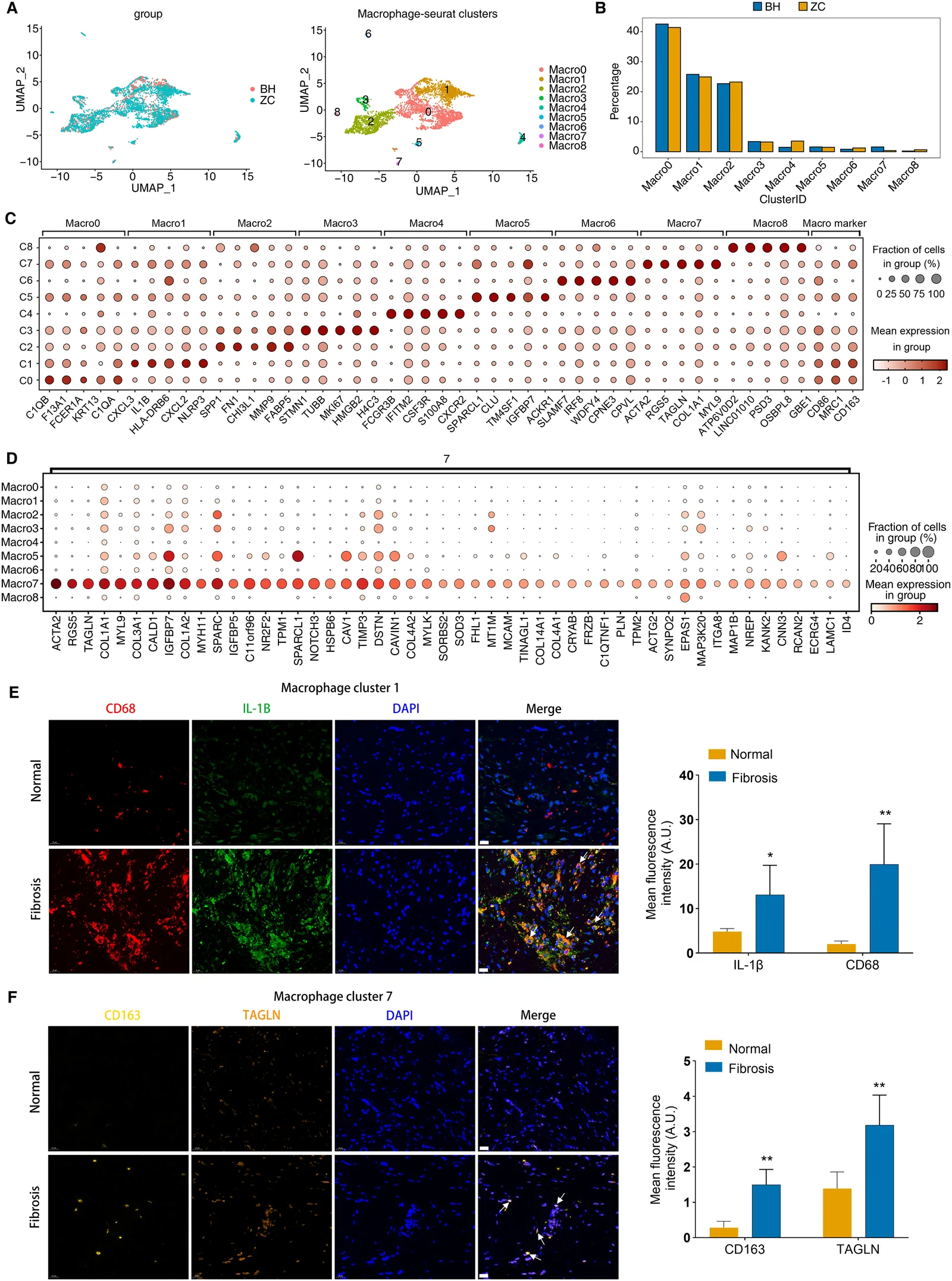
Single-cell transcriptomic profiling of macrophage heterogeneity. **(A)** UMAP plot of 14 clusters (left) assigned to nine macrophage subpopulations (right) based on the marker gene expression. **(B)** Proportions of nine macrophage subpopulations in the BH and ZC groups. **(C)** A dot plot displaying the top marker genes and canonical macrophage markers (CD86, MRC1, and CD163) for each macrophage subpopulation. **(D)** A dot plot displaying the top 50 marker genes in the macro7 subpopulation. **(E)** Immunofluorescence staining showing the localization and expression of CD68 and IL-1β in the Macro1 subpopulation from normal and fibrotic tissues, with their mean fluorescence intensity quantified. **(F)** Immunofluorescence staining showing the localization and expression of CD163 and TAGLN in the Macro7 subpopulation from normal and fibrotic tissues, with their mean fluorescence intensity quantified. For scRNA-seq, three patients with paired tissues were analyzed. For immunofluorescence, five patients with paired tissues were analyzed, and five randomly selected fields per section were quantified. Data are presented as mean ± SD. Statistical significance was determined using a paired Student’s t-test. *p < 0.05, **p < 0.01.

**Figure 6:**
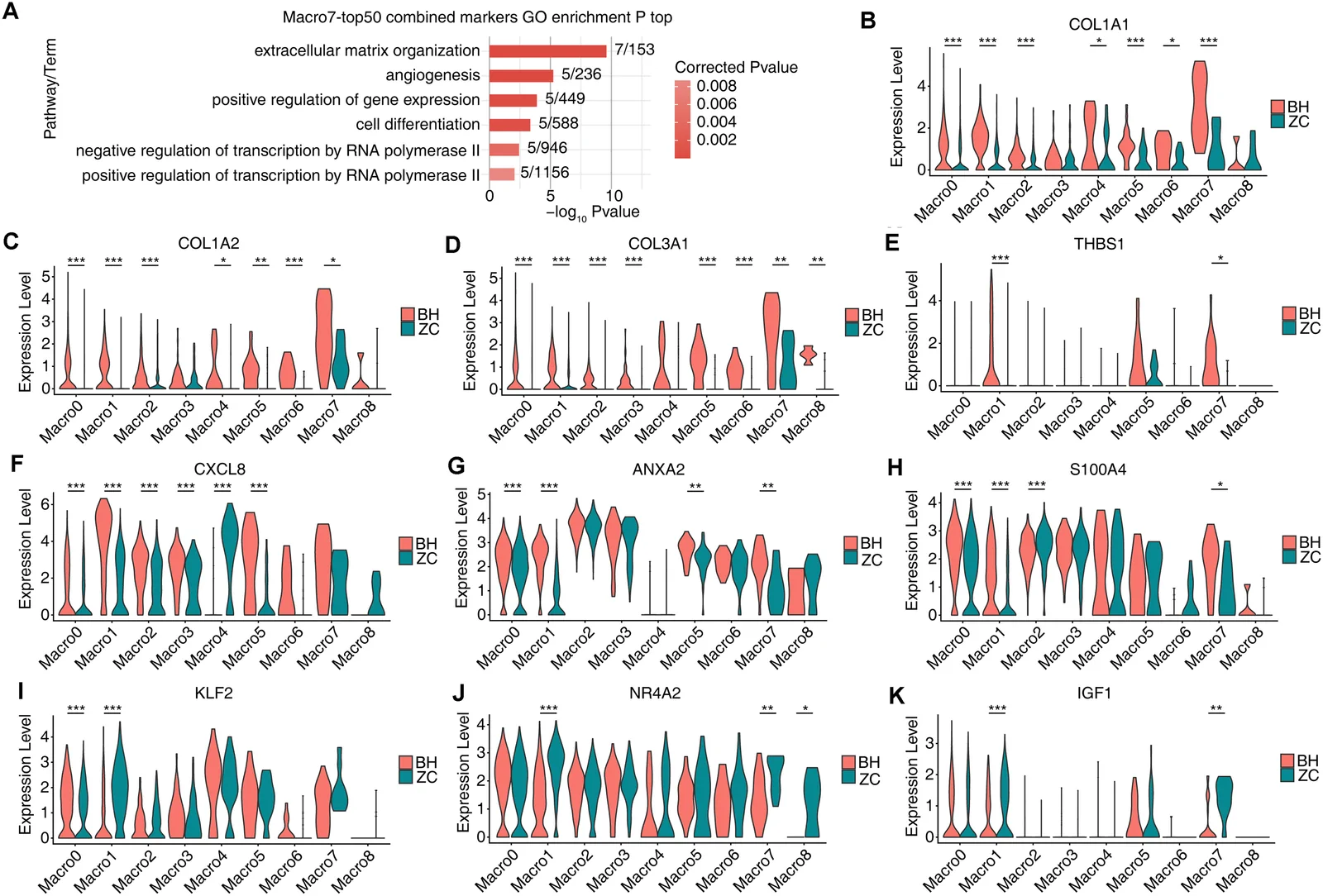
Transcriptomic features and GO enrichment of macrophage subclusters. **(A)** The GO terms enriched by the top 50 marker genes in the Macro7 subpopulation. **(B–K)** Violin plots showing the expression level of COL1A1, COL1A2, COL3A1, THBS1, CXCL8, ANXA2, S100A4, KLF2, NR4A2, and IGF1 in nine macrophage subpopulations between the BH and ZC groups. Three patients with paired scar (BH) and normal (ZC) tissues were analyzed. Statistical significance was determined using the Wilcoxon rank-sum test. *p < 0.05, **p < 0.01, ***p < 0.001.

### Cell-cell interaction patterns in the BH and ZC groups

Based on the sequencing data from single cells, we performed cell-cell communication analysis using Cellchat to reveal the connections between different fibroblast subclusters and other cells. The overall number and strength of communication were enhanced in the BH group compared to those in the ZC group (**Figure 7A**). Potential interactions between fibroblast subclusters and other cells were observed in both groups (**Figure 7B**). The interactions between fibroblasts and other cell types increased during the disease. Next, we employed heat maps to display the interactions between different cells. A variety of fibroblasts were distinctly active and had increased communication with epithelia, macrophages, and SMC, especially the FB0 and FB3 subclusters (**Figure 7C**). We further compared the proportion of information flow of important signaling pathways for cell interaction in each group. The intensity of signaling pathways such as NRXN, SELE, and NCAM was significantly upregulated in the BH group, whereas those of SPP1, CLEC, and EDN were downregulated (**Figure 7D**).

**Figure 7:**
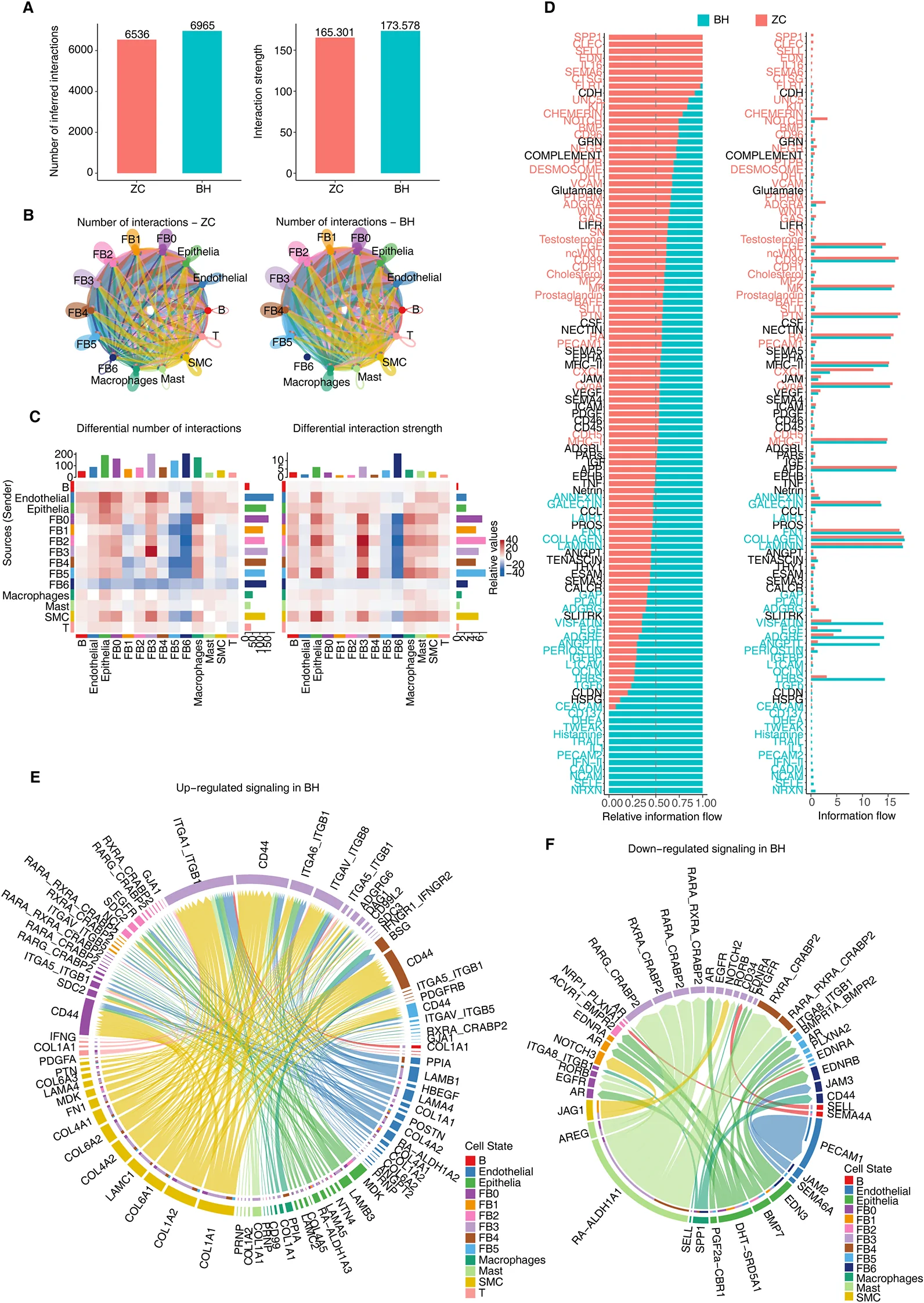
Analysis of cell-cell interaction using CellChat. **(A)** Bar plots revealing the total number and strength of intracellular communication in the BH and ZC groups. **(B)** Global communication maps of seven fibroblast subclusters and other cell types for the BH and ZC groups. **(C)** Total interaction number or strength between different fibroblast subclusters and other cell types in the BH and ZC groups. **(D)** Signaling pathway differences in cell communication between the BH and ZC groups. **(E, F)** Circle plots showing upregulated and downregulated signaling pathways that are important for communication between fibroblast subclusters and other cell types for the BH group. Three patients with paired scar (BH) and normal (ZC) tissues were analyzed. CellChat analysis was performed as described in Methods.

We also used a network to exhibit complex intercellular signaling. As shown in **Figures 7E, F**, there were more upregulated ligand-receptor signaling pathways than downregulated pathways in the BH group. In these upregulated pathways, mast cells, macrophages, endothelial cells, and SMCs mainly used the ligand COL1A1/COL1A2 to transmit signals to the receptors ITGA1_ITGB1, ITGAV_ITGB8, and CD44 in the FB3 subcluster. Macrophages transmit signals to the FB2 or FB4 subclusters through the EREG-EGFR or PPIA-BSG pathways. **Supplementary Figures S5A–K** displays the expression of these key ligands and receptors in different fibroblast subclusters of the BH and ZC groups. Moreover, most of the upregulated ligand-receptor signaling pathways from various cell types to fibroblast subclusters belonged to the LAMININ and collagen pathways (**Supplementary Figure S5L, M**). Among the downregulated signaling pathways, epithelial cell communication to the FB2 subcluster was dependent on the BMP7-ACVR1_BMPR2 pathway and signaled the FB5 subcluster through the BMP7-BMPR1A_BMPR2 pathway. In general, the dense intercellular communication network between fibroblast subclusters and other cell types may be critical for the pathological mechanism of urethral fibrosis.

We further conducted cell-cell communication analysis and observed close connections between different macrophage subclusters and fibroblasts in both the BH and ZC groups (**Supplementary Figure S6**). The Macro7 subcluster showed strong interactions with other macrophage subclusters and fibroblasts in the BH group (**Supplementary Figure S6A**), whereas it did not interact with other macrophage subclusters and fibroblasts in the ZC group (**Supplementary Figure S6B**). The interaction number and strength of the Macro7 subcluster were markedly higher than those of the other macrophage subclusters and fibroblasts (**Supplementary Figure S6C**). Overall, these data indicated that Macro7 may play a potential role in the pathological mechanism of urethral fibrosis.

## Discussion

Urethral stricture is a heterogeneous disease with different etiologies, clinical features, and treatment outcomes (33). Understanding the cellular heterogeneity and regulatory changes in tissues under pathological conditions is critical for developing appropriate therapeutic strategies (34). Although fibroblasts play an important role in intercellular communication in urethral strictures, the heterogeneity of cell subpopulations and regulatory mechanisms in the disease remain unclear (12). In this study, we deconstructed the major cell types in urethral scars and normal tissues through scRNA-seq analysis and further highlighted the functional characteristics of fibroblasts, mast cells, and macrophages in the disease. Compared with the two prior scRNA-seq studies of urethral stricture, our study profiles a larger number of cells (65, 054) and simultaneously analyzes fibroblast, mast cell, and macrophage heterogeneity (11, 12). It also provides evidence for the co-localization of key markers and their potential association with fibrosis. While previous studies focused on single etiologies and specific cell lineages, our integrated SCENIC, scMetabolism, and CellChat analysis provides a more comprehensive framework for urethral stricture.

In this study, preliminary annotation of eight major cell types revealed significant differences in their distributions between the BH and ZC groups. Fibroblasts were markedly enlarged in the BH group, suggesting that this cell type exhibited a relative enrichment in the disease environment. Because the overactivation of urethral fibroblasts is a core biological event that causes urethral strictures, we explored their heterogeneity (35). We classified fibroblasts into seven subclusters based on the marker genes of the cells. Among these, the proportion of the FB3 subcluster was clearly elevated in the BH group. Further, GO analysis showed that the top 100 marker genes of the FB3 subcluster were mainly enriched in the biological processes of cell adhesion and ECM organization. The FB3 subcluster is associated with the development and remission of urethral strictures. Further exploration revealed that the FB3 subcluster displayed different transcriptional and metabolic characteristics in the BH and ZC groups. ETS1, a potential candidate TF, was highly expressed in diseased tissues. Moreover, its target genes were significantly enriched in multiple terms, such as protein polyubiquitination. ETS1 regulates the expression of ECM-related genes, thereby affecting various pathological processes, including fibrosis (36). Increased ETS1 expression has been reported to promote development of lung fibrosis (37). Metabolic pathway-related analyses revealed that the HMOX1-mediated upregulation of porphyrin and chlorophyll metabolism contributed to urethral scarring. Excessive HMOX1 expression causes myocardial injury and fibrosis in the mouse heart, and pharmacological inhibition of HMOX1 ameliorates Adriamycin-induced cardiotoxicity (38). However, an association between porphyrin and chlorophyll metabolism and fibrosis has not been reported. Based on these studies, we hypothesized that targeting the FB3 subcluster may provide new insights into urethral stricture treatment.

scRNA-seq revealed an abnormal increase in mast cell abundance in the BH group. Mast cells are located near fibroblasts, and their abundance is positively correlated with fibrosis severity (39). During lung injury, active mast cells promote collagen production and fibroblast proliferation (40). However, the role of mast cells in urethral fibrosis remains unknown. In this study, we identified two mast cell subclusters. The proportion of the Mast1 subcluster in the BH group was elevated compared with that in the ZC group, whereas the Mast0 subcluster showed the opposite trend. Mast1 subcluster-associated gene markers were distinctly activated in response to copper/zinc ions. Trace elements are involved in various basic metabolic functions, and dysregulation of copper or zinc in cells occur during the development of organ fibrosis in the lungs, further supporting the importance of the Mast1 subcluster in urethral fibrosis (41, 42). Next, we characterized the function and metabolism of the Mast1 subcluster using TF and metabolic pathway analyses. The expression of SMAD3, a potential candidate TF, exhibited the highest upregulation in the Mast1 subcluster and was mainly enriched in the GO terms signal transduction and actin cytoskeleton reorganization. Overrecruited mast cells enhance the ECM by activating the TGF-β1/SMAD2/3 signaling pathway, consequently inducing the development of hepatic fibrosis (43). In the present study, nitrogen metabolism significantly increased in the BH group. Dysregulation of nitrogen metabolism leads to fibrosis in steatohepatitis (44). Although these molecules and their biological functions are involved in the pathological changes associated with fibrosis, their association with urethral fibrosis remains unclear. Therefore, further studies are required to confirm the clinical significance of these results.

We also explored the heterogeneity of macrophages. Macrophages are essential for wound healing and are involved in liver fibrosis (45). Their polarization status determines fibroblast activation and subsequent biological behavior (46). In the present study, we identified nine macrophage subpopulations. Among these, abundance of the Macro1 and Macro7 subpopulations clearly increased in the BH group. Our immunofluorescence staining showed increased IL-1β and TAGLN expression in Macro1 and Macro7 from fibrosis tissues, respectively. IL-1β regulates collagen and fibronectinand facilitates collagen deposition (47). In addition, TAGLN is associated with multiple fibrosis-related proteins and contributes to kidney fibrosis (48). Increased expression of COL1A1, COL1A2, COL3A1, THBS1, and S100A4 was observed in the Macro1 and Macro7 subclusters. These markers have been reported to be associated with several fibrotic conditions (49–52). In the present study, GO enrichment analysis revealed that the top 50 marker genes in the Macro7 subcluster were mainly enriched in ECM organization. Overall, we hypothesized that the Macro1 and Macro7 subpopulations may contribute tourethral fibrosis by modulating ECM organization.

Ligand-receptor crosstalk analysis was also employed to elucidate communication patterns between cells. We observed complex communication patterns between the fibroblast subclusters and other cell types, particularly the FB3 subcluster. Analysis of the upregulated signals in BH samples indicated that CD44 and ITGA-ITGB signals secreted by the FB3 subcluster play important roles in communication. Integrins mediate signal transmission and are involved in inflammatory and fibrotic responses (53). Additionally, the interactions of these signaling molecules in the FB3 subcluster with COL1A1, expressed by mast cells, were increased in BH tissues. The expression of COL1A1, a downstream gene regulated by the TGF-β1 signaling pathway, was high in patients with lung fibrosis (54). Collagen signaling pathways, such as COL4A and COL6A, dominated the upregulated signaling pathways, consequently emphasizing their possible key role in the regulation of fibrosis. In addition, close connections were observed between different macrophage subclusters and fibroblasts in both the BH and ZC groups, indicating a critical role for macrophage-fibroblast crosstalk in the progression of fibrosis. The Macro7 subcluster exhibited robust interactions with other macrophage subclusters and fibroblasts in the BH group but not in the ZC group. This suggests that the Macro7 subcluster plays a potential role in the pathological processes associated with urethral fibrosis. These findings provide new insights into the pathological mechanisms and potential therapeutic targets of urethral fibrosis.

This study identified the major cell types involved in urethral fibrosis based on scRNA-seq analysis, with an emphasis on the diversity of fibroblasts, mast cells, and macrophages and their subtle transcriptional regulatory mechanisms. Although the present study revealed some unrecognized pathological changes in urethral fibrosis, some non-negligible limitations remain. First, this study focused on the characteristics of fibroblasts or mast cells in disease but did not explain the origin and transformation of these cell clusters. Secondly, the causal relationship between these key cell clusters and the progression of urethral fibrosis is unclear; thus, their spatial dynamics in the development of fibrosis warrant further exploration. Finally, the results relied on transcriptomic analysis, and the functions of specific cells lacked experimental confirmation. Given that only three patients were included, the findings of this study should be interpreted with caution, and future studies with multicenter data and larger sample sizes are needed to improve the reliability of the results.

## Conclusion

Collectively, our proposed novel fibroblast subclusters and intercellular communication may contribute to the fibrotic process in urethral stenosis. The expanded FB3 fibroblast subpopulation, Mast1, Macro1, and Macro7 subpopulations may be associated with urethral fibrosis through biological processes such as cell adhesion and ECM organization. These data provide a theoretical resource for studying the pathology of urethral stricture and may facilitate the development of therapeutic measures targeting specific cell types.

## Data Availability

The raw and processed single-cell RNA sequencing data in the study are deposited in the NCBI Gene Expression Omnibus (GEO), accession number GSE347480. The main analysis code is available at Zenodo (DOI: 10.5281/zenodo.22899743).

## Supplementary material

Supplementary Figure 1 Differential analysis of single-cell transcriptional profiles in the BH and ZC groups. **(A)** A bubble plot showing the relative expression of the top three marker genes in 27 cell types. **(B)** A bubble plot showing the number of DEGs for each annotated cell type between the BH and ZC groups. **(C, D)** Heatmaps displaying GO enrichment of DEGs with upregulated and downregulated expression in each cell type. Three patients with paired scar (BH) and normal (ZC) tissues were analyzed. Differential expression was analyzed using the Wilcoxon rank-sum test with Bonferroni correction, as described in Methods.

Supplementary Figure 2 Transcriptional regulation analysis of the fibroblast FB0 subcluster. Violin plots showing the AUC score and expression levels of key TFs in each fibroblast subcluster for the BH and ZC groups, including KLF5 **(A, B)**, KLF6 **(C, D)**, SOX6 **(E, F)**, HOXA13 **(G, H)**, and FOXF1 **(I, J)**. **(K)** A volcano plot showing differentially expressed TFs in the FB0 subcluster between the BH and ZC groups. **(L–O)** Violin plots showing the AUC score and expression level of SMAD5 or CEBPB in each fibroblast subcluster for the BH and ZC groups. **(P)** The TF-target gene network for the FB0 subcluster. The top five TFs with upregulated and downregulated expression were selected. The square represents TF, and the triangle represents the target gene. **(Q)** A heatmap displaying GO biological process enrichment of the target genes of TFs in the FB0 subcluster. Three patients with paired scar (BH) and normal (ZC) tissues were analyzed. Statistical significance for differentially expressed TFs was determined using the Wilcoxon rank-sum test with Bonferroni correction.

Supplementary Figure 3 Comparison of metabolic pathways of the FB2 subcluster between the BH and ZC groups. **(A)** A heatmap showing upregulation of porphyrin and chlorophyll metabolism in the BH group. **(B)** A Venn diagram of the DEGs with upregulated expression in the FB2 subcluster and gene set in porphyrin and chlorophyll metabolism. Three patients with paired scar (BH) and normal (ZC) tissues were analyzed.

Supplementary Figure 4 Comparison of TFs **(A)** and metabolic pathway **(B)** patterns in two subclusters of mast cells. Three patients with paired scar (BH) and normal (ZC) tissues were analyzed.

Supplementary Figure 5 Expression of key ligands and receptors in different fibroblast subclusters from the BH and ZC groups within the intracellular communication assay. **(A–D)** Violin plots showing the expression of key ligands, including COL1A1, COL1A2, EREG, and BMP7. **(E–K)** Violin plots demonstrating the expression of key receptors, including ITGA1, ITGB1, ITGAV, ITGB8, CD44, EGFR, and BSG. **(L, M)** Relative contribution of the ligand-receptor pair of two key signaling pathways (LAMININ and collagen) in the BH group. Three patients with paired scar (BH) and normal (ZC) tissues were analyzed. CellChat analysis was performed as described in Methods.

Supplementary Figure 6 Analysis of cell-cell interaction between nine macrophage subpopulations and fibroblasts. **(A, B)** Global communication maps of nine macrophage subpopulations and fibroblasts in the BH **(A)** and ZC **(B)** groups. **(C)** Differential interaction number or strength between nine different macrophage subpopulations and fibroblasts. Three patients with paired scar (BH) and normal (ZC) tissues were analyzed. CellChat analysis was performed as described in Methods.

Supplementary Figure 7 Patient-level pseudobulk analysis of BH vs ZC. **(A)** Overlap of DEGs identified by single-cell and pseudobulk analyses across eight cell types. **(B)** Concordance of effect sizes between single-cell and pseudobulk analyses. Three patients with paired scar (BH) and normal (ZC) tissues were analyzed. Pseudobulk analysis and statistical tests were performed as described in Methods.
